# Genome Sequence of a Potentially New *Buttiauxella* Species, Strain B2, Isolated from Rhizosphere of Olivillo Trees (*Aextoxicon punctatum*)

**DOI:** 10.1128/MRA.01351-19

**Published:** 2020-02-27

**Authors:** Romina Almasia, Marlene Henríquez, Arturo Levican, Matías Poblete-Morales

**Affiliations:** a Departamento I+D+i, Microagro SpA, Santiago, Chile; b Carrera Tecnología Médica, Facultad de Ciencias, Pontificia Universidad Católica de Valparaíso, Valparaíso, Chile; c Facultad de Ciencias de la Salud, Instituto de Ciencias Biomédicas, Universidad Autónoma de Chile, Santiago, Chile; Indiana University, Bloomington

## Abstract

We announce the draft genome sequence of strain B2, which belongs to a potentially new *Buttiauxella* species, isolated from soil associated with rhizosphere of olivillo trees (*Aextoxicon punctatum*). Its size is 4,967,099 bp, and its G+C content is 49.1%. The genome of strain B2 carries genes related to rhizobacteria that promote the growth of plants.

## ANNOUNCEMENT

The genus *Buttiauxella* belongs to the family *Enterobacteriaceae* and includes Gram-negative motile rods which are facultative, anaerobic, and negative for oxidase (1). It was named in 1982 by Ferragut et al. (1) and currently comprises seven validly accepted species, Buttiauxella agrestis, Buttiauxella brennerae, Buttiauxella ferragutiae, Buttiauxella gaviniae, Buttiauxella izardii, Buttiauxella noackiae, and Buttiauxella warmboldiae (2). They have been isolated from mollusks and snails, as well as from water, food, soil, and human samples (2). The main characteristics that allow differentiating of the species of this genus are DNA-DNA hybridization characteristics and the guanine-plus-cytosine (G+C) ratio, which ranges from 48% to 50% (3). Recently, two potentially new species have been isolated from plants, one of them from the rhizosphere of a chrysanthemum (*Chrysanthemum* sp.) plantation in Brazil (Buttiauxella chrysanthemi sp. nov.) and the other from sedum (Sedum alfredii), a hyperaccumulator plant used for phytoremediation of heavy metal contamination in soil (strain SaSR13) (4, 5). The latter strain significantly improves plant growth, the development of the root, and the accumulation of cadmium in *Sedum alfredii* (5). Furthermore, among isolates from the genus *Buttiauxella*, there have been described phytases that allow the use of inorganic phosphate applicable as a nutritional supplement for animals (6). In fact, Ruangsanka (7), through the analysis of 16S rRNA gene sequences of bacteria associated with bamboo rhizosphere, observed that bacteria with the highest production of soluble phosphate belong to the genus *Buttiauxella*.

*Buttiauxella* sp. strain B2 was isolated from the rhizosphere of olivillo trees (Aextoxicon punctatum) in Cerro Santa Ines, Coquimbo Region, Chile (–32.1632, –71.4949; altitude, 663 m), in August 2015. For isolation, 20 g of roots with adhering soil was resuspended in 180 ml of sterile saline (0.85% NaCl). This mixture was homogenized in a previously sterilized glass blender three times for 1 min each with intermittent steps of placing the glass container in ice for 1 min. An aliquot of 1 ml of the supernatant was submitted to a serial 10-fold dilution in sterile saline. Then, 100 μl of each dilution was inoculated onto solid plates of LB medium (Beckton Dickinson, USA). The agar plates were incubated at 28°C for 72 hours, and single colonies were restreaked onto a new LB plate. The isolate was classified within the *Enterobacteriaceae* family through phenotypic characterization, i.e., Gram-negative, motile rods which were negative for oxidase and positive for oxidation and fermentation of glucose in oxidation-fermentative (OF) medium. Furthermore, the 16S rRNA gene was amplified and sequenced as previously described (8). The sequence obtained (GenBank accession number MN759732) showed an identity of 99.25% with bacteria of the genus *Buttiauxella* according to the online tool EzBioCloud (https://www.ezbiocloud.net).

For genomic DNA extraction, bacteria were grown on LB medium at 28°C for 24 hours under aerobic conditions, and then a single colony was picked and subcultured in 5 ml of LB broth medium, which was incubated overnight at 28°C in a shaker at 200 rpm. DNA was extracted using the Wizard genomic DNA purification kit (Promega Corp., USA) following the manufacturer’s instructions for Gram-negative bacterial cells. The library was prepared with the TruSeq Nano DNA kit, and genomic sequencing was carried out with the Illumina MiSeq platform with 2 × 150-bp paired-end (PE) reads by Macrogen, Inc. (Seoul, South Korea). A total of 11,149,234 reads were obtained, and they were quality checked and filtered with the FastQC v0.11.5 application using the default parameters. K-mer analysis was performed in order to estimate coverage, heterozygosity, and genome size with Jellyfish v2.2.10 and GenomeScope (http://qb.cshl.edu/genomescope/). Then, *de novo* assembly was performed by using the filtered reads with SOAPdenovo2 v2.04. All of these basic bioinformatic analyses were performed by Macrogen, Inc. A total of 75 contigs were obtained (*L*_50_, 8; *N*_50_, 189,923 bp) with an average depth of 126.74×, and the estimated length of the chromosome was 4,967,099 bp with a G+C content of 49.1%.

Functional annotation of the assembled genome was performed with the Rapid Annotations using Subsystems Technology (RAST) server v2.0 (9) (http://rast.nmpdr.org/rast.cgi) via RASTtk (10). A total of 4,676 genes were identified, i.e., 4,610 coding sequences (CDS), 63 RNAs, and 3 rRNAs. PGAP annotation was also performed by default when genomes were deposited in NCBI (11). However, the annotations in RAST and PGAP were not compared, because that is beyond the scope of this study.

**FIG 1:**
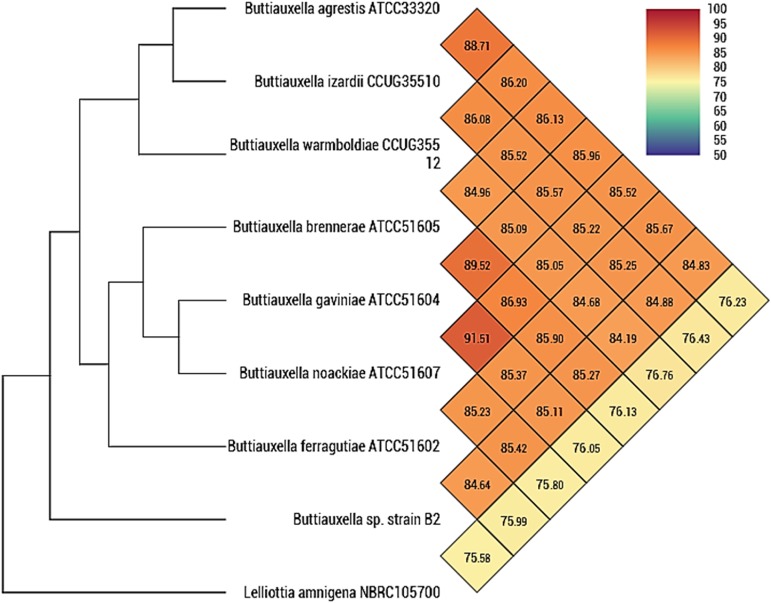
Heatmap showing the average nucleotide identity by orthology (OrthoANI) between strain B2 and all the type strains of the genus *Buttiauxella* calculated with OAT software with Lelliottia amnigena used as the outgroup.

In order to determine the genomic identity of strain B2, the orthologous average nucleotide identity was performed to measure the overall similarity between genome sequences using the OrthoANI v0.93.1 online tool available at EzBioCloud (https://www.ezbiocloud.net/tools/orthoani) (12). Low identity values were obtained between strain B2 and the type strain of the genus *Buttiauxella* (Fig. 1).

The genome of *Buttiauxella* sp. strain B2 encoded two genes classified in the category of plant-prokaryote by the U.S. Department of Energy (DOE) project. That project aims to determine the pangenomes of 100 to 200 species of soil- or plant-associated prokaryotes (https://jgi.doe.gov/core-pangenomes-soil-plant-associated-prokaryotes/). Furthermore, it encoded five genes associated with the production of plant hormones (Table 1). On the other hand, a strong capacity of phosphate solubilization was observed when culturing the bacteria in solid National Botanical Research Institute's phosphate growth medium (NBRIP) as previously described (13). Moreover, gene clusters associated with solubilization of phosphate were found in the genome by using the bacterial antiSMASH tool v3.0 with the default parameters (Table 1) (14, 15). Therefore, the presence of plant-associated genes, as well as the observed capacity of phosphate solubilization, warrants further studies to determine whether this bacterium possesses symbiotic interactions with the host species *Aextoxicon punctatum* and a biotechnological potential.

**TABLE 1:** Genes associated with plant growth and phosphate-solubilizing traits (antiSMASH tool) present in the genome of *Buttiauxella* sp. strain B2

Trait and category	Subcategory	Subsystem	Role (EC no.)	RAST features or no. of contigs/start-stop position/strand (antiSMASH tool)
Plant growth promotion				
Secondary metabolism	Plant hormones	Auxin biosynthesis	Tryptophan synthase alpha chain (4.2.1.20)	fig|6666666.374956.peg.200
			Anthranilate phosphoribosyltransferase (2.4.2.18)	fig|6666666.374956.peg.197
			Tryptophan synthase beta chain (4.2.1.20)	fig|6666666.374956.peg.199
			Monoamine oxidase (1.4.3.4)	fig|6666666.374956.peg.75
			Phosphoribosylanthranilate isomerase (5.3.1.24)	fig|6666666.374956.peg.198
Miscellaneous	Plant-prokaryote DOE project	Single-rhodanese-domain proteins	Thiosulfate sulfurtransferase GlpE (2.8.1.1)	fig|6666666.374956.peg.4358
			Rhodanese domain protein, enterobacterial subgroup, YceA homolog	fig|6666666.374956.peg.1859
Phosphate solubilization				
			Oligopeptide-binding protein (AppA)	1/501295-502887/−
			Major phosphate-irrepressible acid phosphatase	3/162750-164021/−
			Periplasmic nitrate reductase	8/129512-131999/+
			Periplasmic nitrate reductase, electron transfer subunit	8/132010-132462/+
			Quinoprotein glucose dehydrogenase	13/74571-76952/−
			Alpha-d-ribose 1-methylphosphonate 5-triphosphate synthase subunit (PhnG)	22/21340-21801/+
			Alpha-d-ribose 1-methylphosphonate 5-triphosphate synthase subunit (PhnH)	22/21798-22382/+
			Alpha-d-ribose 1-methylphosphonate 5-triphosphate synthase subunit (PhnI)	22/22382-23446/+
			Alpha-d-ribose 1-methylphosphonate 5-phosphate C-P lyase	22/23439-24287/+

The genome sequence of strain B2 was compared with all available sequences of *Buttiauxella* spp. by using the OrthoANI standalone software tool that is freely available at http://www.ezbiocloud.net/sw/oat (12). OrthoANI is a robust and fast means of calculating average nucleotide identity for species delineation; it does not require gene-finding or functional annotation processes, allowing simple, reproducible, and standardized procedures (12). Although Lee et al. (12) recommended a 95% to 96% OrthoANI value threshold for species demarcation, the interspecies values between the accepted *Buttiauxella* spp. are even lower, i.e., 84.68% to 91.51% (Fig. 1). Along these lines, OrthoANI values between strain B2 and accepted species ranged from 84.19% to 85.42% (Fig. 1). Therefore, these results support the finding that strain B2 actually belongs to a potentially new *Buttiauxella* species, even though a future polyphasic study is necessary to demonstrate this taxonomic position. Moreover, the presence of genes related to rhizobacteria that promote the growth of plants warrants future studies to determine their *in vivo* effects on the host species.

### Data availability.

This whole-genome sequence project has been deposited in GenBank under the accession number VEXQ00000000. The version described in this paper is version VEXQ01000000. The BioProject number is PRJNA548017, and the BioSample number is SAMN11997788.
